# The Ikaros Transcription Factor Regulates Responsiveness to IL-12 and Expression of IL-2 Receptor Alpha in Mature, Activated CD8 T Cells

**DOI:** 10.1371/journal.pone.0057435

**Published:** 2013-02-26

**Authors:** Eric T. Clambey, Bernard Collins, Mary H. Young, Jens Eberlein, Alexandria David, John W. Kappler, Philippa Marrack

**Affiliations:** 1 Integrated Department of Immunology, University of Colorado Anschutz Medical Campus, Aurora, Colorado, United States of America; 2 Howard Hughes Medical Institute, National Jewish Health, Denver, Colorado, United States of America; 3 Department of Anesthesiology, University of Colorado Anschutz Medical Campus, Aurora, Colorado, United States of America; 4 Mucosal Inflammation Program, University of Colorado Anschutz Medical Campus, Aurora, Colorado, United States of America; 5 Department of Biochemistry and Molecular Genetics, University of Colorado Anschutz Medical Campus, Aurora, Colorado, United States of America; 6 Barbara Davis Center for Childhood Diabetes, University of Colorado Anschutz Medical Campus, Aurora, Colorado, United States of America; 7 Department of Medicine, University of Colorado Anschutz Medical Campus, Aurora, Colorado, United States of America; 8 Department of Pharmacology, University of Colorado Anschutz Medical Campus, Aurora, Colorado, United States of America; Saint Louis University School of Medicine, United States of America

## Abstract

The Ikaros family of transcription factors is critical for normal T cell development while limiting malignant transformation. Mature CD8 T cells express multiple Ikaros family members, yet little is known about their function in this context. To test the functions of this gene family, we used retroviral transduction to express a naturally occurring, dominant negative (DN) isoform of Ikaros in activated CD8 T cells. Notably, expression of DN Ikaros profoundly enhanced the competitive advantage of activated CD8 T cells cultured in IL-12, such that by 6 days of culture, DN Ikaros-transduced cells were 100-fold more abundant than control cells. Expression of a DN isoform of Helios, a related Ikaros-family transcription factor, conferred a similar advantage to transduced cells in IL-12. While DN Ikaros-transduced cells had higher expression of the IL-2 receptor alpha chain, DN Ikaros-transduced cells achieved their competitive advantage through an IL-2 independent mechanism. Finally, the competitive advantage of DN Ikaros-transduced cells was manifested *in vivo*, following adoptive transfer of transduced cells. These data identify the Ikaros family of transcription factors as regulators of cytokine responsiveness in activated CD8 T cells, and suggest a role for this family in influencing effector and memory CD8 T cell differentiation.

## Introduction

CD8 T cells control primary and secondary infections by multiple pathogens [1]. Following T cell activation, CD8 T cells acquire multiple effector functions, including cytokine production, cytolytic activity, and the capacity to become long-lived CD8 memory T cells. CD8 T cell differentiation to effector and memory cell fates is heavily influenced by the nature and duration of T cell stimulation and the inflammatory milieu [2]. The molecular determinants that regulate mature CD8 T cell activation and differentiation are incompletely defined.

The Ikaros family of transcription factors includes the Ikaros, Aiolos, Helios, Eos and Pegasus proteins [3]. Ikaros, the founding member of this family, functions to activate and repress transcription, and plays a central role in hematopoietic development, lineage decisions and as a tumor suppressor [3]. These transcription factors have a high degree of conservation in both their N-terminal DNA-binding zinc fingers and C-terminal dimerization zinc fingers [4]. Optimal DNA binding requires homo- or heterodimerization of Ikaros family members each containing DNA-binding domains [4]. Given the similarity of these proteins and their ability to dimerize, this family has a high degree of genetic redundancy. Splice isoforms which lack DNA-binding domains, but retain the dimerization domains, can function as dominant negative molecules, effectively interfering with the function of multiple family members [4], [5]. Naturally occurring dominant negative variants can be generated by alternative splicing, and can be detected in healthy cells at low levels [6], and in malignancies where Ikaros-family loss of function is thought to be critical for progression to malignancy [7].

The Ikaros family has important roles in developing and mature T cells. For example, neonatal Ikaros-deficient mice have a complete defect in fetal thymocyte development, and adult Ikaros-deficient animals have thymocyte development skewed towards CD4 T cells [8], [9]. Ikaros also regulates T cell receptor signal transduction and T cells with reduced Ikaros activity have enhanced TCR signaling and activation [10]. Ikaros family members are also regulated during T cell activation and proliferation, with Ikaros colocalizing with DNA replication machinery during activation-induced proliferation [10] and Helios recently identified as a protein upregulated during T cell activation and proliferation [11]. In mature CD4 T cells, Ikaros regulates multiple processes including Th2 differentiation and cytokine expression (e.g. IL-2 and IL-10) [12], [13], [14], [15]. Recent studies have identified roles for the Ikaros family in regulatory T cells (Helios, Eos) and Th17 cells (Aiolos) [16], [17], [18], [19]. In addition, Helios was identified by a network analysis approach as a gene whose expression was elevated in CD8 T cells during chronic infection [20].

While Ikaros regulates CD8α expression in thymocytes, its actions, and those of related proteins, in mature CD8 T cells remains poorly characterized [21]. Here we show that mature CD8 T cells express multiple Ikaros family members. Further, we used expression of a naturally occurring, dominant negative variant of Ikaros to selectively interfere with the function of the Ikaros family following T cell activation. These studies identify a prominent role of the Ikaros family in regulating cytokine responsiveness of mature CD8 T cells.

## Materials and Methods

### Mice

C57BL/6 and B6.SJL-*Ptprc^a^ Pep3^b^*/BoyJ (CD45.1+) mice were obtained from The Jackson Laboratory (Bar Harbor, ME). OT-I TCR transgenic mice (specific for the ovalbumin peptide SIINFEKL) [22] were provided by Dr. T. Potter (University of Colorado Denver, USA) and P14 TCR transgenic mice (specific for the lymphocytic choriomeningitis virus gp33 peptide, KAVYNFATM) were from Dr. P. Ohashi (University of Toronto, Toronto, Canada) [23]. OT-I mice were crossed to B6.SJL-*Ptprc^a^ Pep3^b^*/BoyJ mice to generate OT-I.CD45.1 mice. OT-I mice were used for the source of all T cells unless noted otherwise. All mice were maintained in a pathogen-free environment in the Biological Resource Center, National Jewish Health, and used in accordance with institutional and federal guidelines. The animal protocol was approved by the Institutional Animal Care and Use Committee of National Jewish Health (under Animal Welfare Assurance Policy A3026-01, IACUC protocol AS2538-07-10) and is in accordance with the National Institutes of Health guidelines for use of live animals.

### Flow Cytometry and Antibodies

Cells were subjected to flow cytometric analysis on a CyAn (Dako) or an LSRII (Becton Dickinson). Antibodies (BD PharMingen) included: CD8 (53-6.7), CD25 (PC61 or 7D4), CD44 (IM7), CD62L (MEL-14), CD69 (H1.2F3), CD90.1 (Thy1.1; Ox-7) and CD122 (TM-β1). 7-aminoactinomycin D (7-AAD) or LIVE/DEAD® Fixable Dead Cell Stain (Invitrogen) were used to identify viable cells in most analyses.

### Cell Culture

Total, unpurified splenocytes (20×10^6^ cells/mL) from OT-I transgenic mice were incubated with cognate peptide (1 µg/mL) for 90 minutes at 37°C (peptides from The Molecular Resource Center, National Jewish Health), after which cells were extensively washed using at least a ten-fold excess of volume to remove any unbound peptide from the culture. On day 1, these same unpurified splenocyte cultures were subjected to retroviral spinfection (2 hours, 2000 rpm at 30°C) in complete medium plus 10% fresh fetal bovine serum, retrovirus and polybrene (8 µg/mL). Because retrovirus transduction requires cells that are actively proliferating, the vast majority (>98%) of retrovirally-transduced cells in these cultures are CD8+ T cells, both 24 and 72 hours post-transduction. On day 2, cells were cultured in fresh media containing various cytokines (1×10^6^ cells/mL). Cultures were counted, analyzed for transduction and phenotype, and passed into media with fresh cytokine every two days. Recombinant cytokines included: hIL-15 and mIL-2 (R & D Systems) at 20 ng/mL; mIL-12 (eBioscience) at 5 ng/mL. For IL-2 neutralization, cultures were treated with 20 µg/mL of isotype control antibody (20LC) or blocking IL-2 antibody (S4B6); S4B6 potently reduced IL-2 (20 ng/mL) driven proliferation (not shown). Retrovirally transduced cells were defined as CD8 T cells expressing Thy1.1, with less than 1% Thy1.1+ in mock-transduced cultures. For all culture experiments in which cultures had a high rate of cell death (cultures done in the absence of additional cytokines and in IL-12), retrovirally transduced cells were defined as live (by exclusion of either 7AAD or the LIVE/DEAD dead cell stain), CD8+ T cells expressing Thy1.1. The Ikaros-deficient JE131 mouse cell line, originally published in [24], was kindly provided by Dr. Susan Winandy (Boston University, Boston, MA, USA).

### DNA Constructs

A plasmid containing the Ik6 dominant negative isoform of Ikaros was kindly provided by Dr. Katia Georgopoulos [4], and a retrovirus expressing dominant negative Helios (Δ49–285) was kindly provided by Dr. Christopher Klug [25]. Retroviruses expressing flg-tagged Ik6 were generated by standard cloning methods into MSCV-IRES-Thy1.1 (MiT) and MSCV-IRES-GFP (MiG) plasmids.

### Retrovirus Preparation & Transduction

Ecotropic retroviruses were generated by co-transfection (Lipofectamine 2000, Invitrogen) of 4∶1 ratio of retroviral DNA and retroviral helper plasmid (pCL-Eco) [26] into Phoenix-Eco cells (from Dr. Garry Nolan). Supernatants were harvested 2–3 days post-transfection, clarified, and concentrated by 16-hour centrifugation (∼4500×g) in Nalgene Oak Ridge FEP Tubes (Fisher Scientific), with resulting pellet resuspended in 1/10^th^ original volume. For MiT-based retroviruses, the viral concentrate was treated for 1 hour with phospholipase C (phosphatidylinositol-specific from *Bacillus cereus*, Sigma) at 37°C to remove GPI-linked proteins, including Thy1.1 from the surface of retroviral particles to ensure detection of productive retroviral transductants.

### Western Blot Analysis

Retrovirally transduced cells (magnetically enriched for Thy1.1+ events, Miltenyi Biotec) or cell lines were collected and whole cell extracts were generated using E1A lysis buffer [27]. PAGE was performed with one million cell equivalents loaded per well on 4–12% Bis-Tris NuPage Gel (Invitrogen), followed by transfer to PVDF membrane. Membranes were blotted with an anti-Ikaros antibody (E-20, Santa Cruz Biotech) followed by an AlexaFluor647-conjugated chicken anti-goat antibody. Results were imaged on a Bio-Rad Versadok 4000 MP and images cropped using Quantity One software.

### Gene Expression Profiling

CEL files from the Gene Expression Omnibus repository (GEO) were analyzed using Genespring software (v11.5). Intensity values for the human T cell dataset GSE23321 (Affymetrix HuGene-1_0-st) were computed using the RMA16 algorithm [28]. Probesets not expressed in at least one of the groups were removed by filtering on a ‘detection above background’ (DABG) p-value cutoff of 0.05. The GSE10239 dataset (Affymetrix MG430_2.0) was normalized using the RMA (robust multi-array analysis) algorithm [29]. The dataset consists of samples from four groups: naïve LCMV-specific TCRtg P14 cells (n = 3), adoptively transferred P14 cells which were FACS-purified 4.5 days after primary LCMV Armstrong infection into Klrg1int (n = 3) and Klrg1hi (n = 3) subpopulations and P14 memory T cells 60–120 days after primary LCMV Armstrong infection (n = 3). For analysis of effector T cells, the Klrg1int and Klrg1hi datasets were grouped together (Eff, n = 6). Present/absent calls for the MG430_2.0 and MG-U74 chipsets were generated utilizing the MAS5 implementation of Genespring. Filtering RMA-normalized data based on present/absent calls generated by the MAS5 algorithm before statistically testing has the propensity to improve the ratio of true versus false positives [30]. Differentially expressed genes were identified after removing low-intensity probesets (lowest 20 percent) using one-way ANOVA (p<0.05) with a false discovery rate cutoff at p<0.05 (Benjamini-Hochberg method) and minimal fold change of 2. Note that the analysis of the human and mouse microarray datasets required separate methods for normalizing the data, either using the RMA (robust multi-array analysis [31]) algorithm for the murine microarray chipset MG430_2.0, or RMA16 for the more recent Affymetrix human microarray GSE23321 (Affymetrix HuGene-1_0-st).

### Statistical Analysis

Statistical analysis was done using either a paired t test or a 2-tailed unpaired t test, as indicated, comparing control transduced and DN Ikaros transduced cells using Prism 4.0c (GraphPad).

### Software

Flow cytometric data were analyzed using FlowJo (TreeStar, Inc.), with data displayed as histograms, with log10 scales (from 10^0^ to 10^4^). Histogram overlays are routinely graphed as “% of maximum events” or “% max”, which allows for comparison of the distribution of events between samples that may contain different cell numbers (e.g. in the case of control transduced samples characterized by high rates of cell death). Samples collected on CyAn were subjected to compensation after collection. Data analysis and plotting were done with Microsoft Excel and Prism 4.0c (GraphPad).

## Results

### The Ikaros Family of Transcription Factors is Expressed in Mature CD8 T Cells

**Figure 1:**
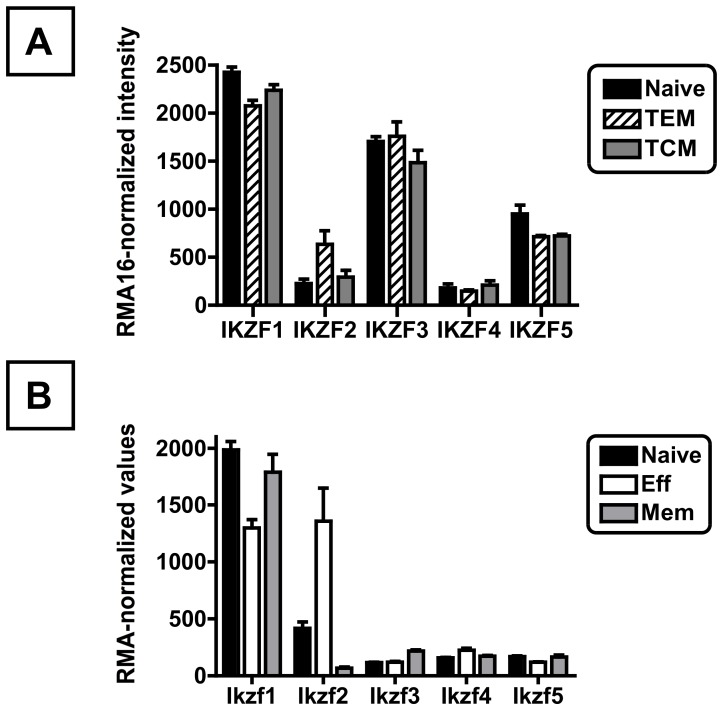
Gene expression profiles of Ikaros family members in mature CD8 T cells derived from Gene Expression Omnibus repository (GEO) datasets. (A) Gene expression profile of human CD8 T cell subsets, including naïve (TN), effector memory (TEM) or central memory CD8 T cells (TCM) with data from GSE23321. (B) Gene expression profile of mouse CD8 T cell subsets, including naïve, effector (Eff) or memory CD8 T cells (Mem) with data from GSE10239. Differential regulation of murine Ikzf1 and Ikzf2 gene expression in P14 cells after LCMV infection with data from GSE10239. Eff expression values are the combined average of array data from sorted Klrg1int or Klrg1hi-expressing P14 cells 4.5 days after LCMV Armstrong infection as in Methods. To allow comparison of microarray hybridization intensities across different samples, microarray data were normalized using standard normalization techniques, either the RMA16 (A) or RMA (B) algorithms to normalize two different generations of Affymetrix microarrays. For both human and mouse datasets, all Ikaros family members are significantly expressed above background (DABG p-values <0.05). Gene names refer to the following gene products: Ikzf1, Ikaros; Ikzf2, Helios; Ikzf3, Aiolos; Ikzf4, Eos; Ikzf5, Pegasus.

To date, there is limited information on Ikaros family members in CD8 T cells. During analysis of published microarray analyses of mouse and human CD8 T cells, however, we noted that all five Ikaros members are expressed at a detectable level, with expression in naïve, effector and memory CD8 T cells in both human and mouse (Fig. 1A,B). In addition, in both humans and mice, there is a trend towards reciprocal regulation of Ikaros (Ikzf1) and Helios (Ikzf2), with Helios mRNA modestly increased in effector or effector memory cells relative to either naive or memory CD8 T cells (Fig. 1A–B). While future studies will be required to investigate Ikaros family mRNA splicing and protein expression in CD8 T cells, these data demonstrate that mature CD8 T cells express Ikaros family members, and suggest that the Ikaros family may influence CD8 T cell function.

### Expression of Dominant Negative Ikaros has Little Effect on the IL-15 or IL-2 Driven Growth of Primary CD8 T Cells, and does not Interfere with Cytokine-driven Differentiation Cues

**Figure 2:**
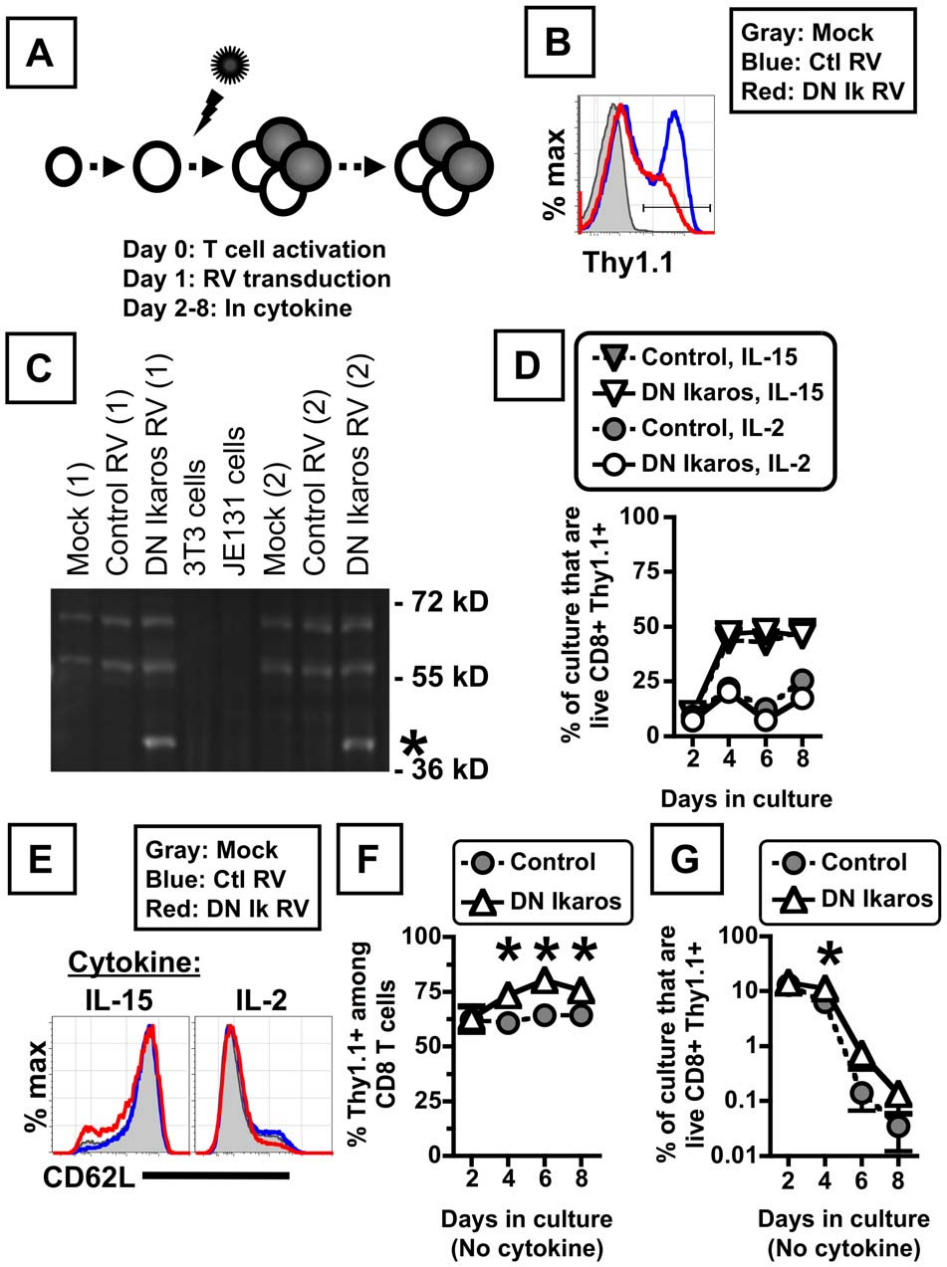
Experimental setup to investigate the consequence of ectopic expression of dominant negative Ikaros on activated CD8 T cells. (A) Schematic of experimental method to analyze the consequence of forced expression of dominant negative (DN) Ikaros on mature, activated CD8 T cells. Retroviral transduced cells, detected by Thy1.1 expression, are depicted in gray. (B) Thy1.1 expression as measured by flow cytometry on activated, retrovirally transduced CD8 T cells at day 8 in culture with IL-2. Histograms are gated on live, CD8+ events in mock (gray), control RV (blue) or DN Ikaros-transduced cultures (red). (C) Western blot analysis of Ikaros expression in peptide-activated, P14 TCR transgenic T cells transduced with control or DN Ikaros (from two independent cultures, 1 and 2). The smaller Ik6 isoform ∼37 kDa is indicated by asterisk. 3T3 fibroblasts and the JE131 Ikaros-deficient thymoma serve as negative controls for Ikaros expression. (C) Relative abundance of retrovirally transduced CD8 T cells cultured in either IL-15 or IL-2. Data indicate the percentage of the culture that are live, transduced cells (defined as CD8+ Thy1.1+) following culture in IL-15 or IL-2, beginning at day 2. Cells were transduced with either control (filled symbols) or DN Ikaros-expressing retrovirus (open symbols), with data representative of two independent experiments. (E) CD62L expression on live, CD8+ (mock, gray) or CD8+ Thy1.1+ events (using gate in panel B) in control (blue) or DN Ikaros (red) transduced cells cultured in IL-15 (left) or IL-2 (right). (F,G) Relative abundance of retrovirally transduced CD8 T cells cultured in the absence of exogenous cytokines, as measured by the percentage of CD8 T cells that are transduced (F) or the percentage of the culture that are live, transduced cells (defined as 7AAD negative, CD8+ Thy1.1+ events) (G). Cells were transduced with either control (gray filled circles) or DN Ikaros-expressing retrovirus (open triangles). Each data point indicates the mean percentage ± SEM) at 2 to 8 days post-activation (cells transduced on day 1 post-activation), with data from four independent experiments and statistically significant differences (p<0.05), determined by paired t test, indicated by asterisk.

Given the expression of multiple Ikaros family members within mature CD8 T cells, and the known genetic redundancy within these gene products, we sought to investigate the contribution of the Ikaros gene family to the properties of mature CD8 T cells. To do this, we used retroviral transduction in vitro to express a previously described dominant negative variant of Ikaros (the Ik6 isoform, DN Ikaros) in activated primary CD8 T cells (experimental design depicted in Fig. 2A). Retrovirally-transduced cells were identified by Thy1.1 expression (encoded within the bicistronic retroviruses used here), with DN Ikaros transduced cells routinely having a modestly reduced expression of the Thy1.1 marker gene relative to control retrovirus transduced cells (Fig. 2B). The Ik6 isoform is known to heterodimerize and to inhibit DNA binding of multiple Ikaros family members [4]. Following transduction, mixed cultures of transduced and untransduced CD8 T cells were placed into different cytokine conditions, and the relative abundance and properties of retrovirally-transduced cells (either control or DN Ikaros-transduced) were analyzed. Western blot analysis for Ikaros protein peptide-activated CD8 T cells identified expression of two major species of endogenous Ikaros (∼50–55 kDa, <72 kDa), with DN Ikaros transduced cells also having robust expression of the smaller Ik6 isoform (∼37 kDa, indicated by asterisk) (Fig. 2C).

To analyze the consequence of antagonizing the function of Ikaros family members within CD8 T cells, we first studied DN Ikaros-transduced cell dynamics and phenotype in either IL-15 or IL-2 cultures, conditions that can differentially induce central memory CD8 T cells or effector CD8 T cells in vitro [32]. Ectopic DN Ikaros expression had little to no effect on proliferation or survival of the cells under these conditions (Fig. 2D). DN Ikaros-transduced cells were comparable to control-transduced cells in size, granularity, and expression of CD8 alpha (CD8α) and CD44 upregulation (data not shown). Moreover, DN Ikaros-transduced cells had the same CD62L expression profile as control-transduced cells, with IL-15 cultured cells expressing high levels of CD62L and IL-2 cultured cells expressing low levels of CD62L, regardless of DN Ikaros expression (Fig. 2E). When cells were cultured in the absence of exogenous cytokines (i.e. cytokine withdrawal), DN Ikaros-transduced cells had a modest advantage relative to non-transduced cells, as revealed by an increase in the percentage of DN Ikaros-transduced cells in the culture over time (Fig. 2F–G). Future studies will be required to determine if the modest advantage of DN Ikaros-transduced cells during cytokine withdrawal results from cell-intrinsic changes or from changes in the secretion or response to either pro-survival factors (e.g. IL-2) or pro-apoptotic factors (e.g. IFN-gamma signaling via Stat1). These data demonstrate that expression of DN Ikaros in activated CD8 T cells does not profoundly skew proliferation, survival or differentiation cues in multiple culture conditions.

### DN Ikaros Expression in CD8 T Cells Increases the Magnitude and Duration of CD25 (IL-2Rα) Expression

**Figure 3:**
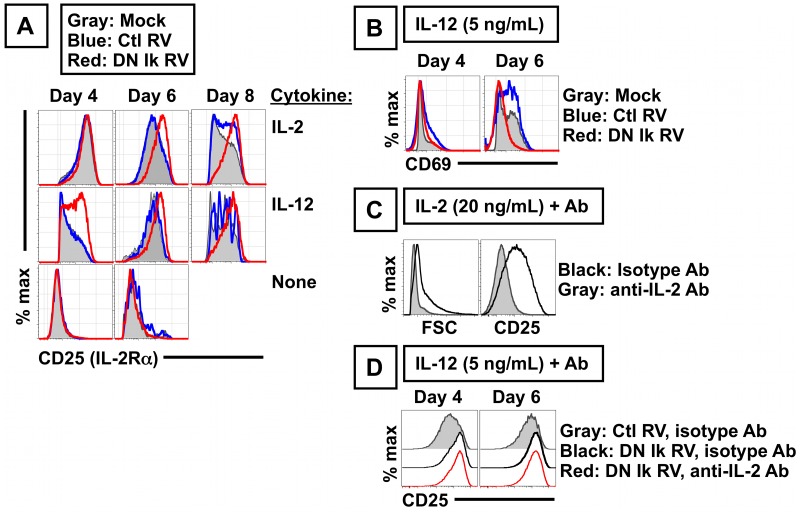
CD8 T cells expressing DN Ikaros have enhanced expression of CD25 (IL-2 receptor alpha chain) in an IL-2 independent manner. (A) Relative abundance of CD25 in CD8 T cells determined by flow cytometry, analyzing cells that were mock transduced (gray), transduced with control vector (blue, Thy1.1+ cells in MiT transduced cultures) or with DN Ikaros retrovirus (red, Thy1.1+ cells in MiT-DN Ikaros transduced cultures). Cells were cultured in mIL-2 (20 ng/mL) (top), mIL-12 (5 ng/mL) (middle) or no exogenous cytokine (bottom). (B) DN Ikaros expression does not enhance CD69 expression in activated CD8 T cells cultured in mIL-12 (5 ng/mL). Data representative of two (IL-2) to five (IL-12) independent experiments. (C) Anti-IL2 antibody effectively neutralizes IL-2 in vitro, as measured by decreased cell size (forward scatter, FSC) or decreased CD25 following culture of activated CD8 T cells in IL-2 (20 ng/mL), comparing treatment with either an isotype control antibody (black) or an anti-IL2 antibody (gray). (D) DN Ikaros-transduced cells cultured in IL-12 (5 ng/mL) have enhanced CD25 expression in an IL-2 independent manner. Data show CD25 expression in DN Ikaros-transduced cultures at day 4 or 6 of culture, in cells treated with isotype control (black) or anti-IL2 antibody (red), relative to cultures treated with an isotype antibody (gray); results representative of two independent experiments.

Although DN Ikaros-transduced cells did not manifest a growth advantage when cultured in IL-2, DN Ikaros-transduced cells had enhanced expression of the IL-2 receptor alpha chain (CD25) (Fig. 3A). DN Ikaros expression promoted earlier CD25 expression on a per cell basis, and more sustained CD25 expression, following culture in either IL-2 (Fig. 3A, top row) or IL-12 (Fig. 3A, middle row), two culture conditions associated with CD25 upregulation. DN Ikaros expression was insufficient to induce CD25 in conditions where CD25 was not already induced (e.g. absence of exogenous cytokines, Fig. 3A, bottom row). The enhanced expression of CD25 was not due to prolonged T cell activation, since another T cell activation marker, CD69, was not increased (Fig. 3B). Preliminary studies indicate that despite the elevated levels of CD25 in IL-12 cultures, there is no difference in intracellular bcl-2 levels in cells transduced with DN Ikaros compared to control transduced cells (not shown).

A primary stimulus that upregulates CD25 expression is IL-2 [33]. Since Ikaros is a repressor of IL-2 [12], [13], we tested the possibility that DN Ikaros-transduced cultures had enhanced IL-2 production, which in turn, upregulated CD25 expression through a positive feedback loop. To test this, we sought to investigate the affect of an IL-2 neutralizing antibody on the phenotype of DN Ikaros-transduced cells. First, in control experiments, we tested the ability of an IL-2 neutralizing antibody to inhibit recombinant IL-2 in vitro; anti-IL-2 antibody was capable of limiting proliferation, and impairing IL-2 driven increases in cell size and CD25 upregulation (Fig. 3C). In parallel experiments, we tested the effects of the anti-IL-2 antibody on CD25 expression levels on DN Ikaros-transduced CD8 T cells grown in IL-12. When DN Ikaros-transduced cultures grown in IL-12 in vitro were treated with the neutralizing IL-2 antibody, there was no effect on the expression level of CD25 on DN Ikaros-transduced cells (Fig. 3D). These data suggest that DN Ikaros expression alters the duration and magnitude of CD25 expression in an IL-2-independent manner.

### DN Ikaros Expression in Activated CD8 T Cells Enhances the Competitive Ability of CD8 T Cells in IL-12

**Figure 4:**
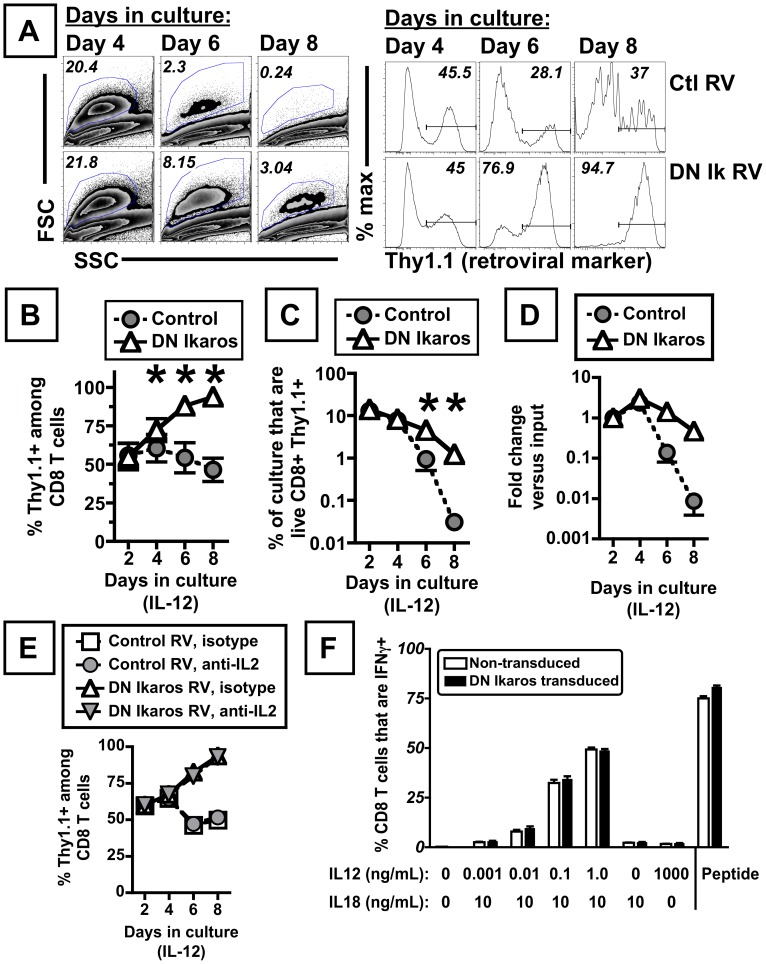
DN Ikaros expression in activated CD8 T cells confers a pronounced advantage for cells cultured in IL-12. (A) Relative abundance of retroviral transduced cells between day 4 to 8 of culture in IL-12, as measured by Thy1.1 expression, in cultures transduced with control (top) or DN Ikaros (bottom) expressing retrovirus. Histograms depict Thy1.1 expression within live (7AAD negative), CD8+ cells as measured by flow cytometry. (B) Data indicate the percentage of CD8 T cells that are transduced from day 2 to 8. (C) Percentage of live, transduced cells (defined as 7AAD negative, CD8+ Thy1.1+ events) between day 2 and 8 of culture in 5 ng/mL of mIL-12. Cells were transduced with either control (gray filled circles) or DN Ikaros-expressing retrovirus (open triangles). Data depict the mean percentage of live, transduced cells (+/− SEM) within cultures at 2 to 8 days post-activation (cells transduced on day 1 post-activation). Data are from five independent experiments, with seven independent cultures, two of which used sorted cells. (D) Data indicate the fold change in number of live, transduced cells (defined as CD8+ Thy1.1+ cells) following culture in mIL-12 (5 ng/mL), relative to the starting number of transduced cells at day 2 of the culture. Data depict mean ± SEM from two independent experiments. (E) Relative abundance of retroviral transduced cells between day 2 to 8 of culture in IL-12 in the presence of neutralizing IL-2 antibodies in cultures transduced with control or DN Ikaros retrovirus. Data indicate percentage of CD8 T cells that are transduced (Thy1.1+) between day 2 and 8 of culture in 5 ng/mL of mIL-12, where cultures were incubated either with an isotype control or an anti-IL2 antibody. Each data point indicates the mean percentage of live, transduced cells (+/− SEM) within cultures at 2 to 8 days post-activation (cells transduced on day 1 post-activation). Data are from two independent experiments. (F) DN Ikaros expression in activated CD8 T cells does not profoundly alter the relative ability of CD8 T cells to respond to IL-12, as measured by IFN-γ production following IL-12/IL-18 synergistic induction of IFN-γ. Activated CD8 T cells, transduced with DN Ikaros were cultured in IL-2 for 6 days and assayed for IFN-γ production by intracellular cytokine staining. Data depict mean of 2–3 replicates per condition. As a positive control, cells were stimulated with cognate peptide (SIINFEKL at 5 µM). Statistically significant differences (p<0.05) are indicated by asterisk, calculated by paired t-test.

Next we tested whether expression of DN Ikaros might confer enhanced survival or proliferation on activated CD8 T cells in the presence of other cytokines (IL-4, IFN-γ or IL-12) that influence CD8 T cell proliferation or differentiation [34], [35], [36], [37]. Dominant negative Ikaros expression in activated CD8 T cells did not significantly change the dynamics of cell growth or survival in response to either IL-4, which induced robust proliferation of activated CD8 T cells, or IFN-γ, in which CD8 T cells exhibited little proliferation (data not shown). When activated CD8 T cells were cultured in IL-12, control cultures showed limited proliferation and a high amount of cell death (Fig. 4A, top panel). Unexpectedly, DN Ikaros-transduced cells demonstrated a striking competitive advantage relative to non-transduced cells in IL-12 cultures, as revealed by the progressive increase in the percentage of DN Ikaros-transduced cells within these cultures over time (identified by Thy1.1 expression, a surrogate marker of retroviral transduction, Fig. 4A–B). By day 8 of culture, DN Ikaros-transduced cultures routinely contained at least 80% DN Ikaros-transduced cells and there was an overall increase in the percentage of viable cells within the culture relative control-transduced cultures (Fig. 4A–B). Notably, the advantage of DN Ikaros transduced cells relative to control cells was more pronounced in IL-12 cultures than that observed following cytokine withdrawal (Fig. 4C compared to Fig. 2G). IL-12 cultures of either untransduced or vector-transduced cells were characterized by limited cell division and a high rate of cell death, with a three-fold increase in the number of transduced cells at day 4, followed by attrition (Fig. 4D). Based on this, DN Ikaros-transduced cells had a profoundly delayed rate of attrition relative to control or non-transduced cells (Fig. 4C–D).

As noted above, one prominent change in gene expression in DN Ikaros-transduced cells was increased CD25 expression (Fig. 3). Given the potent proliferative and survival cues mediated by IL-2, we tested whether the competitive advantage of DN Ikaros-transduced cells in IL-12 culture was dependent on IL-2. Notably, neutralizing IL-2 in IL-12 cultures had minimal effect on the relative abundance of either control or DN Ikaros-transduced cells, with DN Ikaros-transduced cells again dominating IL-12 cultures over time (Fig. 4E). These data indicate that DN Ikaros-transduced cells achieve an IL-2 independent advantage during culture in IL-12.

The advantage of DN Ikaros-transduced cells in IL-12 could be due to an altered sensitivity to IL-12. To test this, we analyzed the ability of DN Ikaros-transduced cells to respond to a wide range of IL-12 concentrations. IL-12, in combination with IL-18, elicits production of IFN-γ in a dose-dependent manner [38], [39]. DN Ikaros-transduced and non-transduced cells responded comparably to a wide range of concentrations of IL-12 (Fig. 4F), indicating that DN Ikaros expression did not profoundly alter the relative ability of activated CD8 T cells to respond to IL-12.

### Expression of a Dominant Negative Isoform of Helios Confers Enhanced Survival of Activated CD8 T Cells in the Presence of IL-12

**Figure 5:**
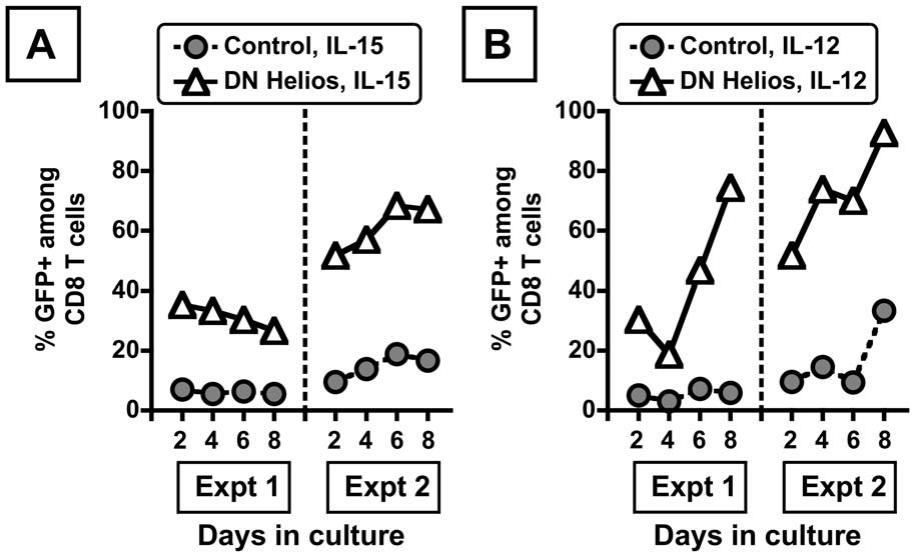
DN Helios expression in activated CD8 T cells confers a competitive advantage for transduced cells cultured in IL-12. Data indicate the percentage of CD8 T cells that are transduced from day 2 to 8, in either IL-15 (20 ng/mL) (A) or IL-12 (5 ng/mL) (B). Cells were transduced with either control (gray filled circles) or DN Helios-expressing retrovirus (open triangles), with retrovirally transduced cells expressing GFP. Data are from two independent experiments. Note that the difference between the control and DN-Helios retroviruses at day 2 reflects a difference in the titers of the retroviral stocks used for transduction.

To investigate whether the enhanced competitiveness of DN Ikaros-transduced cells cultured in IL-12 was a general effect of antagonizing the Ikaros family, we tested the effect of a dominant negative isoform of Helios (HeliosΔ49–285, ref. [25]). While expression of DN Helios had a limited effect on the relative abundance of transduced cells in IL-15 (Fig. 5A), DN Helios expression conferred a more profound competitive advantage to transduced CD8 T cells cultured in IL-12 (Fig. 5B). These data indicate that multiple dominant negative isoforms of the Ikaros family can confer enhanced competitiveness of activated CD8 T cells cultured in IL-12.

### DN Ikaros Expression Confers a Modest Advantage for CD8 T Cells in vivo

**Figure 6:**
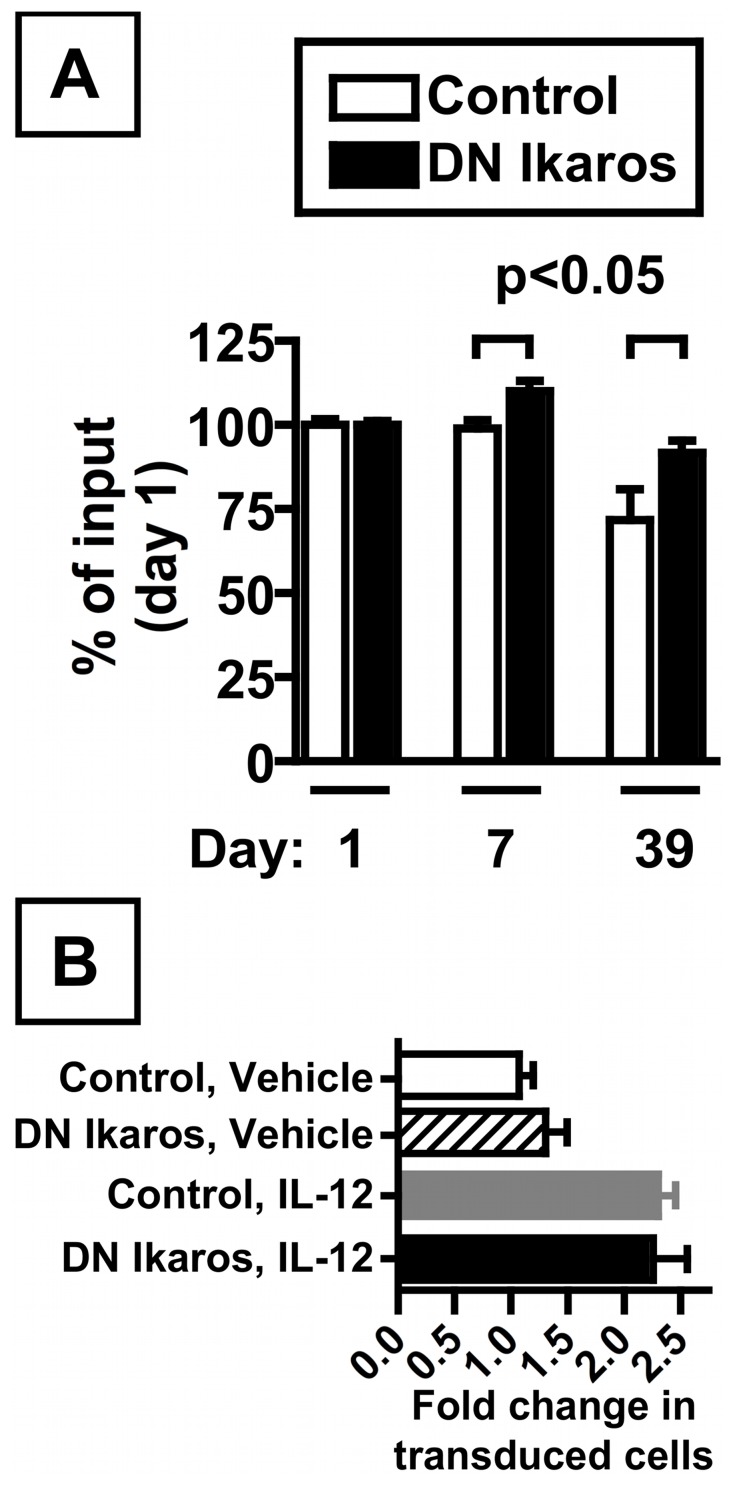
CD8 T cells expressing dominant negative Ikaros have a modest advantage in vivo. (A) Relative abundance of retrovirally transduced cells within a pool of adoptively transferred CD8 T cells containing both transduced and non-transduced cells. Adoptively transferred cells were identified as live, CD8+ MHC class II negative cells that expressed CD45.1, with retrovirally transduced cells further expressing Thy1.1, with abundance measured within the peripheral blood of C57BL/6 recipient mice (which do not express CD45.1 or Thy1.1). Cells were transduced with a control retrovirus (white bar) or a DN Ikaros expressing retrovirus (black bar). To standardize for slightly different starting frequencies, values are standardized relative to day 1 input values for either control or DN Ikaros transduction efficiencies with data showing mean ± SEM with 3–10 mice for control transduced and 6–10 mice for DN Ikaros transduced cells. Mice were bled on day 1, 7, and 39 post-transfer. Statistically significant differences (p<0.05) are indicated, calculated by unpaired t test. (B) Fold change in the abundance of retrovirally transduced cells in C57BL/6J recipient mice from panel A (either control or DN Ikaros transduced) in the blood (defined as the percentage of live, CD8+ MHC class II negative cells that were CD45.1+ Thy1.1+) following injection of either vehicle alone or recombinant IL-12 (1 µg) at day 162 and 165 post-adoptive transfer, with abundance post-treatment measured at day 168 post-transfer. For these studies, at day 1 after adoptive transfer, the frequency of adoptively transferred, retrovirally transduced cells (defined as CD45.1+ Thy1.1+) among CD8 T cells in the peripheral blood was 1.21±0.07% for control and 1.70±0.09% for DN Ikaros transduced cells (mean ± SEM). Among adoptively transferred cells (CD45.1+), the percentage (mean ± SEM) of transduced cells was 42.1±0.7% for control and 49.6±0.5% for DN Ikaros.

To begin to address the in vivo consequence of DN Ikaros expression on CD8 T cell responses, we adoptively transferred DN Ikaros-transduced cells, grown in vitro, into normal C57BL/6J mice and followed their relative competitive ability in vivo (Fig. 6A). By monitoring the relative abundance of transduced cells in peripheral blood, standardized to input frequencies, we found that DN Ikaros-transduced cells had increased abundance relative to control-transduced cells at both day 7 and day 39 post-transfer (Fig. 6A). This initial advantage was not retained long-term, however, with equivalent abundance of DN Ikaros and control-transduced cells by day 160 post-transfer (not shown).

Given the advantage of DN-Ikaros transduced cells in response to IL-12 in vitro, we analyzed the abundance of DN Ikaros-transduced cells following injection of recombinant IL-12 in vivo, a treatment that stimulates CD8 memory T cell proliferation [40]. Relative to vehicle-treated mice, mice injected with IL-12 had an increased prevalence of adoptively transferred cells within the CD8 T cell pool (relative to vehicle-treated mice) (Fig. 6B). However, control and DN Ikaros-transduced cells had comparable increases in their abundance following IL-12 treatment relative to vehicle treated animals (Fig. 6B). These data demonstrate that expression of DN Ikaros confers a modest advantage on CD8 T cells during differentiation and proliferation in vivo.

## Discussion

CD8 T cells have the capacity to achieve distinct phenotypic and functional outcomes [2], [36], [41], [42], [43], [44]. It is now clear that extrinsic cues (including T cell receptor stimulation, cytokines, and cell contact) shape the CD8 T cell response, and that these extrinsic cues are molecularly interpreted by transcription factors [45], [46]. Multiple transcription factors, including Bcl-6 and Bcl-6b, Blimp1, T-bet, Eomesodermin, Id2 and TCF-1 [36], [47], [48], [49], [50], [51], [52], [53], regulate CD8 T cell differentiation.

Ikaros, the best-characterized member of the Ikaros family, is a major hematopoietic transcriptional regulator [3]. Ikaros can both activate and repress transcription, and regulates transcription through multiple mechanisms, including recruitment of chromatin remodeling machinery (e.g. Mi-2β) [54]. Given that Ikaros-family proteins bind to DNA as dimers, heterodimers of full-length Ikaros with a short, non-DNA-binding isoforms results in a molecule incapable of binding to DNA (the basis of the dominant negative approach) [4]. Expression of these dominant negative isoforms are thought to play a critical role in promoting tumorigenesis in both mice and man [55], [56].

To date, little is known about the Ikaros family in mature CD8 T cells, an issue confounded by the role of Ikaros in T cell development, CD8 T cells and T cell receptor signaling [5], [8], [10]. By using retroviral transduction of a dominant negative isoform of Ikaros in activated CD8 T cells, we bypassed the role of Ikaros in development and activation, to study the Ikaros family within activated CD8 T cells. While this analysis is not a genetic deficiency of Ikaros or related family members, our data clearly identify that functional antagonism of the Ikaros-family results in two major alterations: i) enhanced survival of cells exposed to the pro-inflammatory cytokine IL-12 and ii) increased, prolonged expression of CD25. Both of these changes occur in an IL-2-independent manner, an important observation given that Ikaros can transcriptional repress IL-2 in CD4 T cells [12], [13].

IL-12 is a heterodimeric cytokine produced by activated antigen presenting cells during inflammation [57]. IL-12 can facilitate CD8 T cell activation [37], enhance cytotoxic function, IFN-gamma production, and influence effector versus memory fate differentiation [36], [57], [58]. Further, IL-12 treatment enhances CD8 T cell mediated control in mouse models of cancer [59], [60], [61], identifying IL-12 as a possible stimulant to enhance CD8 T cell immunotherapy [62], [63]. Based on our results, Ik6 expression may potentially enhance the efficacy of adoptive immunotherapy in the context of cancer.

At this time, it is unclear why ectopic expression of dominant negative Ikaros-family members specifically enhances the competitive ability of activated CD8 T cells in cultures of IL-12 and not in other cytokines. Although it is possible that Ikaros modifies IL-12 signaling (e.g. Ikaros promotes STAT4 and represses T-bet expression [64], [65]), our data indicate that DN Ikaros-transduced cells do not have significantly altered sensitivity to IL-12. Further, while DN Ikaros expression confers a modest advantage in vivo, IL-12 injection in vivo failed to reveal a sizable advantage of DN Ikaros-transduced cells. This result may be explained in part by the pleiotropic effects of IL-12 injection, which also induces interferon-gamma and IL-15 [40]. An important future question is how DN Ikaros-transduced cells participate during an immune response in vivo, particularly since IL-12 and IL-2 have multiple effects on CD8 T cell differentiation [58], [66], [67], [68], [69], [70]. In addition, the genetic analysis of individual Ikaros family members in CD8 T cells presents an important future goal that awaits the development of new genetic tools (e.g. there are no conditional alleles for Ikaros, which has an important role in T cell development).

The current studies provide an initial investigation of the role of the Ikaros family of transcription factors and their impact on CD8 T cells using a reductionist in vitro system. In these assays, we have used previously established dominant negative constructs to inhibit the Ikaros family, combined with retroviral transduction targeted to peptide-activated CD8 T cells. Although these studies were done using bulk splenocyte culture, retroviral transduction is restricted to peptide-activated, proliferating CD8 T cells since other splenocytes have not received mitogenic stimulation. While we anticipate that the effects of DN Ikaros result from intrinsic alterations within CD8 T cells, further studies will be necessary to conclusively demonstrate that these alterations are mediated strictly through changes within CD8 T cells and that there is no contribution from other cell populations on these phenotypes.

In conclusion, through the use of retroviral transduction we investigated the role of the Ikaros family of transcription factors in mature, activated CD8 T cells. These data define the Ikaros family as potential regulators of cytokine responsiveness, regulating CD25 expression and the competitive ability to survive in pro-inflammatory conditions (e.g. IL-12). Based on these data, we postulate that the Ikaros family will integrate with multiple transcription factors to optimally orchestrate CD8 T cell immunity.
